# Editorial: Maintenance of Genome Integrity: DNA Damage Sensing, Signaling, Repair, and Replication in Plants

**DOI:** 10.3389/fpls.2016.00064

**Published:** 2016-02-15

**Authors:** Alma Balestrazzi, V. Mohan Murali Achary, Anca Macovei, Kaoru O. Yoshiyama, Ayako N. Sakamoto

**Affiliations:** ^1^Department of Biology and Biotechnology “L. Spallanzani”, University of PaviaPavia, Italy; ^2^Department of Plant Molecular Biology, International Centre for Genetic Engineering and BiotechnologyNew Delhi, India; ^3^Plant Breeding, Genetics and Biotechnology Division, International Rice Research InstituteLos Baños, Philippines; ^4^Faculty of Life Sciences, Kyoto Sangyo UniversityKyoto, Japan; ^5^Biotechnology and Medical Application Division, Japan Atomic Energy AgencyTakasaki, Japan

**Keywords:** genome integrity, DNA damage, DNA repair, replication, double strand breaks

Because of their sessile lifestyles, plants are continuously exposed to DNA-damaging agents present in the environment. Although the basic mechanisms of genome maintenance are conserved between animal and plant kingdom, plants also have evolved specific mechanisms to cope with DNA damage. Indeed, studies in past decades have demonstrated the presence of elastic mechanisms in plants. For example, when exposed to DNA damaging agents, plants respond immediately to start repairing the damage, regulating cell proliferation, changing metabolic pathways. Here we are proud to have twelve outstanding articles focus on the maintenance of genome integrity: DNA damage sensing, signaling, repair and replication in plants.

The present e-book is opened by several comprehensive reviews dealing with genomic and extra-genomic DNA maintenance, as well as the role of double strand break (DSB) signaling in plants. In his minireview, Roy provides an upgraded view on the link connecting chromatin structure stability and DNA damage response at the genetic and epigenetic levels, while Amiard et al. present an overview on DSB repair pathways in *Arabidopsis thaliana*, with focus on the signaling of DNA breaks and deprotected telomeres. Stability of genomic DNA, not only in nuclei, but also in organelles is crucial for plant development. In contrast to nuclear genomes, the amount and structural integrity of organellar genomes changes during plant development. It is very interesting to consider why organellar genomes are less stable as the replication and repair machineries are encoded by the nuclear genome, yet the cause of the instability is poorly understood. Oldenburg and Bendich first explain the history of the studies into the size and structure of organellar DNA (orgDNA). They then address the copy number and integrity of orgDNA during plant development. The review continues with an overview of the proteins which are involved in the processes of orgDNA replication, repair, and recombination, and changes in the amount of these proteins during leaf development. It has been observed that plastid DNA (ptDNA) maintenance in grasses differs from that in dicots as it rapidly declines upon light exposure. From these observations, the authors propose the idea that instead of repairing damaged DNA, grasses use a cost-saving involving a loss of ptDNA.

DNA polymerases are crucial for maintenance of genome integrity in organisms. The family X DNA polymerases work in DNA repairs such as base excision repair (BER) and/or DSB repair. Plants are unique in having only one member of this family, polymerase lambda (Polλ). Furukawa et al. showed that the Polλ knockout Arabidopsis (*atpol*λ*-1*) is only mildly sensitive to DSB-inducing treatments, whereas the double-knockouts of AtPolλ and AtLig4 made the plants hypersensitive to DSB compared to each single knockout. These results suggest that the AtPolλ has a role in DSB repair, probably in an AtLig4-independent non-homologous end-joining (NHEJ) pathway. Proliferating cell nuclear antigen (PCNA) is a key component of eukaryotic DNA replication machinery. PCNA usually accompanies the DNA polymerase to gather a specific set of proteins onto the replication fork when replication is disturbed. Cyclin Ds are expressed in G2 and degraded during G2/M transition. When the checkpoint is activated, the degradation of cyclin D is inhibited to arrest cells at G2. Strzalka et al. demonstrated that Arabidopsis PCNAs directly interact with some members of the cyclin Ds in yeast and plant cells, suggesting that PCNAs link the signal of disturbed replication with cell cycle control. Ultraviolet (UV) light has been used to analyze cellular DNA-damage responses. UV induces cyclobutanate pyrimidine dimmers (CPDs) and other damage to DNA, which triggers various cellular responses: DNA repair, cell cycle delay or arrest, and cell death. Thus, UVB in sunlight can confer severe stress to plants, but plants have photorepair enzymes to correct the damage. Takahashi et al. investigated the responses of plant cells irradiated with low or high dose of UVB. UVB irradiated cells showed different reactions, depending on the dose, suggesting that accumulation of CPDs caused by high dose UVB induces formation of single or DSBs, which leads to cell death.

In their research article, Qüesta et al. discuss on the roles of the *DDM1* and *ROS1* genes in UVB-induced DNA repair by using Arabidopsis mutants and a set of analytical measurements. Disruption of these genes had an opposite impact of these two genes, with the *ddm1* mutants accumulating more DNA damage, while the *ros1* mutants showed less amount of DNA damage. Based on experimental work, they hypothesize that the DNA demethylation in the *ddm1* mutant can affect the accessibility of DNA repair systems in this region, while the better performance of *ros1* mutants can arise as a result of increased levels of photolyases. In their research on the MAPK signal transduction in response to aluminum (Al) treatments, Panda and Achary underline the biphasic mode of action of Al-induced DNA damage in *Allium cepa*. They observed that at high concentrations Al induces DNA damage, while at low concentration an adaptive response is present, and hypothesize that the MAPK-DNA repair network is responsible for both actions. The role played by DNA/RNA helicases against the genotoxic effects of abiotic stress is documented. XPB (xeroderma pigmentosum type B) helicases promote nucleotide excision repair (NER) by unwinding double strand DNA at the damaged sites. In the attempt to assess the potential of plant XPB helicases as tools in counteracting adverse environmental conditions, Raikwar et al. carried out an *in silico* and functional characterization of the rice *OsXPB2* gene promoter in response to abiotic stress and hormone-based treatments. Based on its multi-stress responsiveness, the *OsXPB2* promoter represents a promising tool for improving the response of crops to genotoxic stress.

Huefner et al. investigated the short- and long-term impact of high-LET (Linear Energy Transfer) HZE (high atomic weight, high energy) particles vs. low-LET gamma rays on genome stability, using Arabidopsis mutants defective in DNA repair and cell-cycle checkpoint. This study highlights the increased sensitivity of Arabidopsis plants to HZE radiation, revealing the predominant role played by ATR (ATM and Rad3-related) protein kinase compared to ATM (ataxia-telangiectasia mutated) in the response to high-LET radiation. In the accompanying article, Missirian et al. compared the transcriptional response in Arabidopsis seedlings exposed to HZE radiation vs. DSB-inducing agents as gamma rays, bleomycin and mitomycin C. These treatments triggered an intense, short-term DSB-specific repair response which was not detected in plants challenged with conventional stresses. A distinctive feature of the HZE transcriptional response was the early activation of key genes involved in the catabolism of cellular components.

With genome editing being the cutting-edge topic of present days, the article by Cantos et al. deals with the implementation of such tool (zinc finger nucleases, ZFNs) for the identification of appropriate regions for safe gene insertion. By harnessing their ability to induce DSBs at the cutting site, ZFNs trigger the NHEJ or homologous recombination (HR) DNA repair pathways at the targeted site. This study used ZFNs with short DNA recognition domains, able to target multiple sites within the rice genome, and subsequently study the integration patterns of the GUS marker gene, allowing the identification of “safe harbors,” intergenic regions with potential high expression.

The present e-book provides an up-date overview of the ongoing research dealing with different aspects of the DNA damage response in plants, highlighting the complexity of molecular networks involved in genome maintenance. A better understanding of DNA damage accumulation/perception/signaling/repair mechanisms *in planta* is expected to speed up crop improvement through conventional breeding and gene-transfer based techniques.

## Author contributions

AB commented the following articles: Huefner et al., Missirian et al., and Raikwar et al.; AM commented the following articles: Roy, Amiard et al., Qüesta et al., Panda and Achary, and Cantos et al.; KOY commented the following article: Oldenburg and Bendich; ANS commented the following articles: Furukawa et al., Strzalka et al., and Takahashi et al. All authors and read and revised the complete editorial.

## Funding

This work was supported by research fellowship from the Japan Society for the Promotion of Science to KY (13J40017) and partially supported by Grant-in-Aid for Scientific Research to AS (No. 25440147) from the Japan Society for the Promotion of Science. Sponsorship from COST Action CM1201: “Biomimetic Radical Chemistry” is gratefully acknowledged.

### Conflict of interest statement

The authors declare that the research was conducted in the absence of any commercial or financial relationships that could be construed as a potential conflict of interest.

